# Supplementary material for: What is meant by validity in maternal and newborn health measurement? A conceptual framework for understanding indicator validation
Source: PLoS One. 2020 May 29;15(5):e0233969. doi: 10.1371/journal.pone.0233969 (PMC7259779; doi:10.1371/journal.pone.0233969)
Supplement: S1 Table — (DOCX) [file pone.0233969.s001.docx]

S1 Table.

| **What does the concept of “validity” of indicators mean to various stakeholders?** |
| --- |
| The meaning of indicator validity means different things to different people, including by different users, languages, and disciplinary backgrounds.  Three most common understandings of indicator validity mentioned were:   1. The extent to which an indicator is meaningful to progress, leads to change, is worth the measurement effort. 2. The extent to which the measurement of an indicator corresponds to the construct of interest and is a good representation of the real world. If an indicator is being used as a proxy for a construct that is too difficult or expensive to measure, this should be explicitly recognised in the production and interpretation of such indicator. The underlying relationship between the proxy and the concept needs to be clear. 3. Assessment of indicator performance against an objective gold standard. Can be seen as the narrowest, most technical, definition of indicator validity, but it was perhaps the most commonly shared understanding of validity among the various stakeholders. |
| **What types of approaches are considered useful in assessing indicator validity?** |
| 1. Key informants mentioned a shift over time in the methods used to assess indicator validity from a focus on methods such as internal consistency, external consistency, reliability, and cognitive interviewing, toward a focus on diagnostic assessment of validity using a gold standard comparison. Such diagnostic-style validity studies predominantly assess maternal and newborn health indicators derived from population-level surveys. Work on indicators capturing reproductive morbidities and obstetric complications using diagnostic-style validation comparing women’s recall to a gold standard, which showed poor diagnostic validity of these indicators over 20 years ago, was an exception to this trend. Overall, key informants welcomed the increased attention to and funding of assessment of diagnostic/criterion validity, but noted that continued research on other types of validity, including those related to meaning and meaningfulness, should not be neglected. Additionally, several respondents expressed the need for results of more routine elements of survey question development and testing, such as cognitive interviews, to be published more consistently for other researchers and stakeholders to use. 2. Key informants highlighted recent development and use of new indicators, such as those capturing maternal and newborn health financing, policies, and health systems. The production of these indicators faces additional challenges with inaccurate, sub-optimally disaggregated, or non-existent data sources. Some respondents opined that the efforts to understand the accuracy of these indicators are not in fact, in a strict sense, validation studies. Rather, they preferred to use the terms verification or triangulation. 3. A shift away from predominantly measuring maternal and newborn care *contacts* toward incorporating elements of care *content* and *quality*. Studies attempting to adjust indicators of access to care with the receipt of actual evidence-based interventions, or effective coverage, have explored several methods (e.g., individual-record linkage, population-level adjustment) and data sources (e.g., facility surveys, routinely collected facility data, women’s recall of receipt of care content). Key informants also mentioned a related phenomenon of a diminishing focus on population-based surveys as a source of indicator data and increasing attention to exploring the quality and usefulness of routinely collected facility-based data. Such focus was seen as being important for improving data quality and its use by facilities, district, regional and national users. Key informants also advocated for further validation work focusing on indicators of patient-centred care, such as satisfaction and respectful care from women’s perspective, while acknowledging that standard gold-standard diagnostic validity assessments are not suitable to understanding the performance of such indicators. |
