# Supplementary material for: What is meant by validity in maternal and newborn health measurement? A conceptual framework for understanding indicator validation
Source: PLoS One. 2020 May 29;15(5):e0233969. doi: 10.1371/journal.pone.0233969 (PMC7259779; doi:10.1371/journal.pone.0233969)
Supplement: Attachment — Submitted filename: Reviewed Nov 20- PONE-D-19-29431_reviewer-MCH indicator validity paper.pdf [file pone.0233969.s002.pdf]

PLOS ONE

What is meant by validity in maternal and newborn health measurement? A conceptual framework for understanding indicator validation

--Manuscript Draft--

**Manuscript Number:**	PONE-D-19-29431
**Article Type:**	Research Article
**Full Title:**	What is meant by validity in maternal and newborn health measurement? A conceptual framework for understanding indicator validation
**Short Title:**	Conceptual framework for validity of maternal and newborn health indicators
**Corresponding Author:**	Lenka Benova<br>Instituut voor Tropische Geneeskunde<br>Antwerp, BELGIUM
**Keywords:**	Maternal health; newborn health; Measurement; indicators; validation; validity
**Abstract:**	Background<br><br>Rigorous monitoring supports progress in achieving maternal and newborn mortality and morbidity reductions. Recent work to strengthen measurement for maternal and newborn health highlights the existence of a large number of indicators being used for this purpose. The definitions and data sources used to produce indicator estimates vary and challenges exist with completeness, accuracy, transparency, and timeliness of data. This paper presents a conceptual overview on how indicator validity is defined and understood by those who develop and use maternal and newborn health indicators.<br><br>Methods<br><br>A conceptual framework of validity was developed using mixed methods. We were guided by principles for conceptual frameworks and by a review of the literature and key maternal and newborn health indicator guidance documents. We also conducted qualitative semi-structured interviews with 32 key informants chosen through purposive sampling.<br><br>Results<br><br>We categorised indicator validity into three main types: criterion, convergent, and construct. Criterion or diagnostic validity, comparing a measure with a gold standard, has predominantly been used to assess indicators of care coverage and content. Studies assessing convergent validity quantify the extent to which two or more indicator measurement approaches, none of which is a gold-standard, relate. Key informants considered construct validity, or the accuracy of the operationalisation of a concept or phenomenon, a critical part of the overall assessment of indicator validity.<br><br>Conclusion<br><br>Given concerns about the large number of maternal and newborn health indicators currently in use, a more consistent understanding of validity can help guide prioritization of key indicators and inform development of new indicators. All three types of validity are relevant for evaluating the performance of maternal and newborn health indicators. We highlight the need to establish a common language and understanding of indicator validity among the various global and local stakeholders working within maternal and newborn health.
**Order of Authors:**	Lenka Benova
	Ann-Beth Moller
	Kathleen Hill
	Lara M. E. Vaz

Powered by Editorial Manager® and ProduXion Manager® from Aries Systems Corporation

	Alison Morgan
	Claudia Hanson
	Katherine Semrau
	Shams Al Arifeen
	Allisyn C. Moran
**Additional Information:**	
**Question**	**Response**
**Financial Disclosure**<br><br>Enter a financial disclosure statement that describes the sources of funding for the work included in this submission. Review the submission guidelines for detailed requirements. View published research articles from *PLOS ONE* for specific examples.<br><br>This statement is required for submission and **will appear in the published article** if the submission is accepted. Please make sure it is accurate.<br><br>**Unfunded studies**<br>Enter: *The author(s) received no specific funding for this work.*<br><br>**Funded studies**<br>Enter a statement with the following details:<br>• Initials of the authors who received each award<br>• Grant numbers awarded to each author<br>• The full name of each funder<br>• URL of each funder website<br>• Did the sponsors or funders play any role in the study design, data collection and analysis, decision to publish, or preparation of the manuscript?<br>• **NO** - Include this sentence at the end of your statement: *The funders had no role in study design, data collection and analysis, decision to publish, or preparation of the manuscript.*<br>• **YES** - Specify the role(s) played.<br><br>* typeset	This work received support from the Bill & Melinda Gates Foundation.
**Competing Interests**<br><br>Use the instructions below to enter a competing interest statement for this submission. On behalf of all authors,	The authors have declared that no competing interests exist.

disclose any competing interests that could be perceived to bias this work—acknowledging all financial support and any other relevant financial or non-financial competing interests.<br><br>This statement **will appear in the published article** if the submission is accepted. Please make sure it is accurate. View published research articles from *PLOS ONE* for specific examples.<br><br>**NO authors have competing interests**<br><br>Enter: *The authors have declared that no competing interests exist.*<br><br>**Authors with competing interests**<br><br>Enter competing interest details beginning with this statement:<br><br>*I have read the journal's policy and the authors of this manuscript have the following competing interests: [insert competing interests here]*<br><br>* typeset	
**Ethics Statement**<br><br>Enter an ethics statement for this submission. This statement is required if the study involved:<br><br>• Human participants<br>• Human specimens or tissue<br>• Vertebrate animals or cephalopods<br>• Vertebrate embryos or tissues<br>• Field research<br><br>Write "N/A" if the submission does not require an ethics statement.<br><br>General guidance is provided below. Consult the submission guidelines for detailed instructions. **Make sure that all information entered here is included in the Methods section of the manuscript.**	No ethics approval was required. Key informant interviews were conducted about indicators and not human data or human tissue, we did not require an ethics approval. The data in the form of interview notes is available open access under the DOI: https://doi.org/10.17037/DATA.00001403<br><br>Please note there is a second manuscript accepted (currently in production) by PLOS One using the same data. PONE-D-19-12568R1.

**Format for specific study types**<br><br>**Human Subject Research (involving human participants and/or tissue)**<br>• Give the name of the institutional review board or ethics committee that approved the study<br>• Include the approval number and/or a statement indicating approval of this research<br>• Indicate the form of consent obtained (written/oral) or the reason that consent was not obtained (e.g. the data were analyzed anonymously)<br><br>**Animal Research (involving vertebrate animals, embryos or tissues)**<br>• Provide the name of the Institutional Animal Care and Use Committee (IACUC) or other relevant ethics board that reviewed the study protocol, and indicate whether they approved this research or granted a formal waiver of ethical approval<br>• Include an approval number if one was obtained<br>• If the study involved *non-human primates*, add *additional details* about animal welfare and steps taken to ameliorate suffering<br>• If anesthesia, euthanasia, or any kind of animal sacrifice is part of the study, include briefly which substances and/or methods were applied<br><br>**Field Research**<br><br>Include the following details if this study involves the collection of plant, animal, or other materials from a natural setting:<br>• Field permit number<br>• Name of the institution or relevant body that granted permission	
**Data Availability**<br><br>Authors are required to make all data underlying the findings described fully available, without restriction, and from the time of publication. PLOS allows rare exceptions to address legal and ethical concerns. See the PLOS Data Policy and FAQ for detailed information.	Yes - all data are fully available without restriction

A Data Availability Statement describing where the data can be found is required at submission. Your answers to this question constitute the Data Availability Statement and **will be published in the article**, if accepted.<br><br>**Important:** Stating ‘data available on request from the author’ is not sufficient. If your data are only available upon request, select ‘No’ for the first question and explain your exceptional situation in the text box.<br><br>Do the authors confirm that all data underlying the findings described in their manuscript are fully available without restriction?	
**Describe where the data may be found in full sentences. If you are copying our sample text, replace any instances of XXX with the appropriate details.**<br><br>• If the data are **held or will be held in a public repository**, include URLs, accession numbers or DOIs. If this information will only be available after acceptance, indicate this by ticking the box below. For example: *All XXX files are available from the XXX database (accession number(s) XXX, XXX.*).<br>• If the data are all contained **within the manuscript and/or Supporting Information files**, enter the following: *All relevant data are within the manuscript and its Supporting Information files.*<br>• If neither of these applies but you are able to provide **details of access elsewhere**, with or without limitations, please do so. For example:<br><br>*Data cannot be shared publicly because of [XXX]. Data are available from the XXX Institutional Data Access / Ethics Committee (contact via XXX) for researchers who meet the criteria for access to confidential data.*<br><br>*The data underlying the results presented in the study are available*	Data on key informant interviews are currently in the process of being deposited with the LSHTM Data Compass. We foresee being able to send the DOI within two weeks.

from (include the name of the third party and contact information or URL).<br>• This text is appropriate if the data are owned by a third party and authors do not have permission to share the data.<br><br>* typeset	
Additional data availability information:	Tick here if the URLs/accession numbers/DOIs will be available only after acceptance of the manuscript for publication so that we can ensure their inclusion before publication.

Powered by Editorial Manager® and ProduXion Manager® from Aries Systems Corporation

Click here to access/download;Manuscript;20191020_MoNITOR

What is meant by validity in maternal and newborn health measurement? A conceptual framework for understanding indicator validation

Authors

Lenka Benova[1,2], Ann-Beth Moller[3], Kathleen Hill[4], Lara M. E. Vaz[5], Alison Morgan[6], Claudia Hanson[7,8], Katherine Semrau[9], Shams Al Arifeen[10], Allisyn C. Moran[11]

Author affiliations

[1] Faculty of Epidemiology and Population Health, London School of Hygiene and Tropical Medicine, London, United Kingdom

[2] Department of Public Health, Institute of Tropical Medicine, Antwerp, Belgium

[3] UNDP/UNFPA/UNICEF/WHO/World Bank Special Programme of Research, Development and Research Training in Human Reproduction (HRP), Department of Reproductive Health and Research, World Health Organization, Geneva, Switzerland

[4] Maternal Child Survival Program, Jhpiego, Washington DC, USA

[5] Department of Global Health, Save the Children, Washington DC, USA

[6] Nossal Institute for Global Health, University of Melbourne, Melbourne, Australia

[7] Global Public Health, Karolinska Institutet, Stockholm, Sweden

[8] Department of Disease Control, London School of Hygiene and Tropical Medicine, London, United Kingdom

[9] Ariadne Labs; Division of Global Health Equity Brigham & Women's Hospital; Department of Medicine Harvard Medical School, Boston, Massachusetts, USA

[10] Maternal and Child Health Division, International Centre for Diarrhoeal Disease Research, Bangladesh (icddr,b), Dhaka, Bangladesh

[11] Department of Maternal, Newborn, Child and Adolescent Health World Health Organization, Geneva, Switzerland

*Corresponding author. Email: LBenova@itg.be

Abstract

Background

Rigorous monitoring supports progress in achieving maternal and newborn mortality and morbidity reductions. Recent work to strengthen measurement for maternal and newborn health highlights the existence of a large number of indicators being used for this purpose. The definitions and data sources used to produce indicator estimates vary and challenges exist with completeness, accuracy, transparency, and timeliness of data. This paper presents a conceptual overview on how indicator validity is defined and understood by those who develop and use maternal and newborn health indicators.

Methods

A conceptual framework of validity was developed using mixed methods. We were guided by principles for conceptual frameworks and by a review of the literature and key maternal and newborn health indicator guidance documents. We also conducted qualitative semi-structured interviews with 32 key informants chosen through purposive sampling.

Results

We categorised indicator validity into three main types: criterion, convergent, and construct. Criterion or diagnostic validity, comparing a measure with a gold standard, has predominantly been used to assess indicators of care coverage and content. Studies assessing convergent validity quantify the extent to which two or more indicator measurement approaches, none of which is a gold-standard, relate. Key informants considered construct validity, or the accuracy of the operationalisation of a concept or phenomenon, a critical part of the overall assessment of indicator validity.

Conclusion

Given concerns about the large number of maternal and newborn health indicators currently in use, a more consistent understanding of validity can help guide prioritization of key indicators and inform development of new indicators. All three types of validity are relevant for evaluating the performance of maternal and newborn health indicators. We highlight the need to establish a common language and understanding of indicator validity among the various global and local stakeholders working within maternal and newborn health.

Introduction

Globally, the latest estimates indicate that 295,000 maternal deaths occurred in 2017, 2.5 million newborns died in 2018, and 2.6 million stillbirths occurred in 2015.[1-3] Tackling this burden has been prioritised in national, regional and global actions, with ambitious targets set for maternal and newborn survival and well-being.[4, 5] A range of indicators are currently used at global, regional, national and sub-national levels to monitor the progress toward these goals, including the state of maternal and newborn health and well-being, as well as the health systems and care processes thought to influence health outcomes (e.g. coverage of care, quality of care, equitable access to and quality of care). Various maternal and newborn health initiatives have produced core indicator lists and a recent effort to map these various indicators found a rapidly expanding number of indicators numbering over 140.[6] Data sources, methods and definitions for estimating these indicators vary and change over time, and additional challenges exist with completeness, accuracy, transparency, and timeliness of available data.

For indicators to track progress, they must be measurable and clearly defined, accurate, reliable, valid, useful, relevant, accessible, specific, and time-bound.[7] The performance of indicators used for global monitoring along these dimensions is of crucial concern. Within the field of maternal and newborn health, work on measuring and improving validity of currently used indicators and indicators under development is a key part of this agenda.[8-11] Assessing the scientific robustness of indicators in the field of maternal and newborn health goes back several decades, along with development of measurement methods. More recently, several high-profile global efforts to identify and prioritise the most relevant maternal and newborn health indicators for consistent and up-to-date tracking of progress have resulted in additional research on indicator validity.[12, 13]

Given the amount of ongoing work to strengthen measurement for maternal and newborn health, increased coordination and harmonization of efforts are essential.[14] Maternal and newborn health are inextricably linked and it is important that measurement efforts address both maternal and newborn health, capture stillbirths, and other perinatal outcomes. In 2015, the World Health Organization (WHO) launched the Mother and Newborn Information for Tracking Outcomes and Results Technical Advisory Group (MoNITOR), which functions as a Technical Advisory body to the WHO on matters of measurement, metrics, and monitoring of maternal and newborn health for the Departments of

Maternal, Newborn, Child and Adolescent Health and Reproductive Health and Research.[15, 16] The purpose of MoNITOR is to provide clear, independent, harmonized, and strategic advice for global and country stakeholders engaged in maternal and newborn health measurement and accountability. This paper is a result of research commissioned and chaired by the MoNITOR Secretariat to provide global guidance.

Objective

The objective of this paper is to present a range of perspectives on how validity of maternal and newborn indicators is defined and understood by those who develop and use these indicators. We do not aim to do so exhaustively; rather, we concentrate on identifying common conceptual and methodological themes and provide examples of different types of validation research approaches. We focus primarily on indicators related to the Sustainable Development Goals (SDGs),[5] the Global Strategy for Women's, Children's, and Adolescents' Health,[17] Every Newborn Action Plan,[18] and Ending Preventable Maternal Mortality[19] and consider maternal and newborn health indicator validation work in countries of all income levels. However, examples are taken mainly from validation research in low- and middle-income country (LMIC) settings, as that is where the double burden of maternal and newborn morbidity and mortality as well as uncertainties regarding data quality concentrate. This paper is a part of a larger body of work led by MoNITOR to develop implementation support tools on 1. measuring validity of maternal and newborn health indicators; 2. prioritising indicators best suited for monitoring progress in various settings; 3. improving indicator usefulness and uptake by the various global and national stakeholders; and 4. identifying gaps that require additional research. These implementation support tools will also include an online tool to facilitate indicator use and interpretation.

Materials and Methods

We developed this paper iteratively, guided by principles for conceptual frameworks defined by Miles and Huberman.[20] We conducted a review of the published literature,[21, 22] a review of key indicator guidance documents, interviews with key informants, and discussions during the May and November 2018 meetings of MoNITOR and several meetings with MoNITOR’s co-chairs.

We used purposive sampling to identify key informants until thematic saturation was achieved. We interviewed 32 respondents from a variety of backgrounds using a semi-structured interview guide covering five themes: the meaning of indicator validity, methodological approaches to assessing validity, acceptable levels of indicator validity, gaps in validation research, and recommendations for addressing these gaps. Interviews were conducted by LB in English between December 2017 and November 2018 and ranged between 45 and 90 minutes. Detailed notes were taken in shorthand during the interviews, transcribed and expanded immediately following the interview. Written materials and relevant publications were received from several respondents after the interview. The full results of the key informant interviews are reported in a separate paper.[23]

We reviewed the literature with a focus on identifying various study designs relevant to indicator validation within the field of maternal and newborn health. We used a combination of text and MeSH terms related to the concepts of 1. Validation (validation, validity, reliability, sensitivity, specificity, verification, concordance, area under the curve, receiver operating curve), 2. Maternal and newborn health (maternal, pregnancy, antenatal, childbirth, peripartum, intrapartum, labour, newborn, neonatal, postpartum, postnatal, perinatal, obstetric, stillbirth), and 3. Indicators (indicator, estimate) and searched Medline, Embase, and Global Health databases on March 16, 2018 for English language articles published since 1990. Further, we used key informant recommendations of publications and reports to complement the search results. We used the key informant interviews and literature to iteratively construct a framework of typologies of validation studies and provide examples of various types of indicator validation work, including the language used to describe such work.

validity is never defined...

For the purpose of this paper, an indicator is a quantifiable characteristic of a defined population which has a standard definition.[24, 25] We limit our consideration to indicators related to the health status and the health care of women and newborns during pregnancy, childbirth and the postnatal period. We aimed to synthesise the various perspectives on understanding and assessing validity of maternal and newborn health indicators obtained from the literature and key stakeholder interviews and to characterise these approaches using a common language to aid efforts to achieve standard measurement language. To help characterize the various approaches used to assess validity of maternal and newborn health indicators, we classified the key types of maternal and newborn health indicators currently in use. For the purpose of this paper, we categorize indicators (Figure 1) using a

framework adapted from Moller and colleagues [6] into the following key domains of maternal and newborn health indicators:

1. Health system – includes human and financial resources, policies, guidelines, mechanisms, and information flows.
2. Access to and availability of care - refers to accessibility of care to users, availability of health facilities, services and essential supplies and equipment.
3. Care coverage - indicators of the extent to which care is used (e.g. antenatal care and newborn care).
4. Care content and quality - includes care content (elements of care delivered as part of care processes) and person-centeredness of care.
5. Impact – refers to the long-term effects on health status, including morbidity and mortality.

Fig 1. Key domains of maternal and newborn health indicators

Results

Three main types of validity of maternal and newborn health indicators were identified from the existing literature and during key informant interviews (S2 Table). These types broadly map onto the social science definitions of criterion, convergent, and construct validity. We describe each type of indicator validity in detail, giving examples of indicators and published studies, with a focus on approaches and measurement methods used to assess validity levels.

Key terms in indicator validity

Validity is an element of measurement and refers to the level of scientific robustness of an indicator with respect to how well it captures a phenomenon or concept of interest.[26] In this paper, we focus on the overall meaning of validity, that is, the extent to which an indicator correctly captures an underlying maternal and newborn health phenomenon.[27] An appraisal of an indicator's validity therefore requires theoretical clarity about the concept that the indicator is intended to measure. An assessment of an indicator's validity should be done in conjunction with an assessment of its reliability, and potentially also the feasibility of its production.

A. Criterion validity: Assessment of criterion validity, also referred to as diagnostic validity, examines whether the operationalization or measurement of a construct behaves as expected. A common way to examine criterion validity is to compare a measurement with a “gold-standard” or reference standard.

B. Convergent validity: Assessment of convergent validity examines the extent to which one measurement is similar to (converges with) other measurements to which it should be related, based on a common underlying construct (i.e. assessment of different methods of capturing the same construct). New or indirect measures are sometimes referred to as surrogate or proxy indicators.

C. Construct validity: An assessment of construct validity examines whether a given measurement technique accurately reflects the construct it is intended to measure.

Reliability captures the extent to which results are repeatable; in other words, how well the method is able to achieve similar measurement over repeated efforts.[24] Studies looking at reliability also use the terms consistency, agreement, and concordance; studies assessing reliability of measures over time also use the terms decay/deterioration (of recall), and repeatability. The four scenarios of the combination of high/low criterion validity and reliability of a measurement are visualised in Figure 2. The center of the bullseye represents the truth or the gold standard against which criterion validity is assessed while the dots represent data points.[28] As can be seen in the scenarios, consistent (reliable) measurement may or may not be accurately capturing the “truth” or gold standard, while consistently valid measurement (hitting the bullseye) may still result in broad variations in estimates (limited reliability).

Fig 2. Visual representation of criterion validity and reliability of measurement

Criterion validity

Studies assessing criterion validity seek to understand the accuracy of a method of measurement compared to a “gold” or reference standard. Assessment of criterion validity measures the extent to which a current or proposed method of estimates for an indicator accurately reflects an objective truth. Within maternal and newborn health, studies of criterion validity have predominantly assessed indicators of care coverage and content, but have also been used to assess impact indicators. Examples of studies assessing criterion validity of maternal and newborn health indicators are shown in Table 1.

Tab 1. Examples of studies assessing criterion validity

Indicator type and measure	Examples of studies (reference standard and setting)
**Impact**<br><br>Prevalence of maternal morbidities or obstetric complications (e.g. haemorrhage, pre-eclampsia/eclampsia, labour dystocia, prolapse), prevalence of severe maternal morbidity or near miss	Women’s self-report compared to:<br>- Biomarkers and/or medical diagnoses as captured in clinical notes in samples drawn from:<br>o Indonesian hospitals[29],<br>o Ghana hospital[30],<br>o A tertiary hospital in Brazil[31],<br>o Maternity hospitals in Bolivia[32].<br><br>Women’s self-report compared to:<br>- Clinical examination findings from a community survey in Egypt[33].
**Care coverage**<br><br>Coverage of maternal and newborn care interventions, skilled provider at birth, caesarean section rate	Women’s self-report compared to:<br>- Observations during antenatal, intrapartum and postnatal care from<br>o Hospitals in Kenya[34, 35],<br>o Health facilities in Mozambique[36].<br><br>Women’s self-report compared to:<br>- Medical records from medical booklets and HMIS: China through population-based survey[37].
**Care content and quality**<br><br>Immediate initiation of breastfeeding, newborn resuscitation	Women’s self-report compared to:<br>- Observations of care in hospitals in Tanzania, Bangladesh, Nepal[38]<br>- Medical records/facility registers in hospitals in Tanzania, Bangladesh, Nepal[38].

Key informants noted that a substantial portion of recent work on assessing criterion validity has focused on indicators of care coverage and content captured in household surveys such as the Demographic Health Surveys (DHS) and Multiple Indicator Cluster Surveys (MICS).[39, 40] Munos and colleagues discuss many considerations and elements of diagnostic-style (criterion) validity studies related to assessing the validity of care coverage indicators based on data from population-level surveys.[41] A

common approach to assessing validity of women's recall of specific events or care content is to compare women's recall (captured during an exit interview or sometime later during a home visit) against a "gold standard" based on direct observations of care or, less commonly, care elements documented in a facility register or patient record. The most important quantitative metrics used by assessments of criterion validity are summarised in Table 2.

Tab 2. Commonly used measures of criterion validity (adapted from [41, 42])

Measure	Definition, calculation, meaning
**Individual-level validity**	
Sensitivity	The percentage of individuals with the outcome/characteristic of interest who were correctly classified as such.
Specificity	The percentage of individuals without the outcome/characteristic of interest correctly classified as such.
Percent agreement or Accuracy	The percentage of individuals who were correctly classified, i.e. for whom the outcome/characteristic of interest being measured is a match to the gold-standard comparison.
Positive predictive value	The probability that an individual who reported having an outcome/characteristic of interest truly had it.
Negative predictive value	The probability that an individual who did not report having an outcome/characteristic of interest truly did not have it.
Area under the receiver operating characteristic curve (AUC)	Plot of sensitivity versus 1-specificity. A value of 1 means a perfect match, 0.5 a random guess. For binary measures, this is the average of sensitivity and specificity.
Other, less commonly used, measures include likelihood ratio[31] and efficiency[30].	
**Population-level validity**	
Inflation factor (IF) or Test to Actual Positive (TAP) ratio	Ratio of the population prevalence based on the measure being assessed in comparison with the true prevalence based on the gold standard.[43] This measure expresses the extent to which the true population prevalence of the indicator is under- or over-estimated, given the sensitivity and specificity of the measure under consideration and the true population prevalence. It is possible for an indicator to show low individual-level accuracy but good population-level accuracy.

Some of the limitations of these predominantly facility-based criterion validity studies include limited generalisability, additional assumptions required to assess the extent of bias affecting population-level estimates, and issues with high coverage of routine care elements, which lead to sample sizes too small to calculate specificity. In addition, maternal and newborn health indicators based on population-level surveys have a two- to five-year recall period. Indicator validity is dependent on the ability of women to recall an event, which may be affected by length of time since the event. Only a few studies have assessed criterion validity based on length of the recall period since pregnancy and childbirth; many report substantial issues in the ability to ensure high follow-up rates and found some deterioration in the accuracy of women's recall as the length of recall period increases.[29, 31, 35, 36]

Despite the numerous metrics to statistically assess criterion validity, there is no consensus on what thresholds indicate acceptable or good indicator validity levels. We present examples of pre-specified cut-offs provided by studies assessing validity of indicators based on women's recall collected during population-based surveys (Table 3). It is important to note that most studies focus solely on assessing validity of indicator numerators. The validity of an indicator's denominator also has implications for the validity of the overall indicator, but has been less commonly evaluated. This is particularly important for indicators where the denominator is the population in need of an intervention. Decades of work to try to define the need for caesarean section as a denominator for a caesarean section rate indicator (including setting benchmark levels of caesarean section rates for all births irrespective of need used as a denominator) have led to the conclusion that the population of women in need of a caesarean section must be defined locally based on the epidemiological profile and context.[44, 45] Similarly, ongoing work to define appropriate denominators of newborns in need of targeted interventions such as resuscitation face a similar challenge since the population of newborns in need of resuscitation may vary based on different context and settings, e.g. be higher in referral compared to primary facilities.[46]

For health systems indicators, the validity of indicators capturing the existence of specific policies is sometimes referred to as "verification". Methods for such research might include a Ministry of Health representative reporting on policies, compared to the "gold standard" of policy existence as a ratified document, assessed through a document review.[47] Existence of a policy, however, does not guarantee its rollout or implementation, merely its existence.

Tab 3. Examples of pre-specified acceptable validity levels

**Source**	**Indicator types**	**Metric and Level**
Ronsmans 1996[42]	Maternal morbidity, obstetric complications	Fairly accurate if sensitivity and specificity >80%; high specificity is very important for rare outcomes to limit over-reporting of actual prevalence
Liu et al. 2013[37]	Coverage and content of antenatal, delivery and postnatal care	Sensitivity/specificity:<br>- Low <0.33<br>- Moderate 0.33-0.66<br>- High >0.66<br>AUC – overall validity “high” if AUC>0.67 (otherwise moderate/low)<br>Population-level bias<br>- Small if 0.8<TAP ratio<1.2<br>- Moderate 0.5<TAP ratio<1.5<br>- Large <0.5 TAP ratio >1.5
Stanton et al. 2013[36]	Maternal and newborn health interventions during peripartum period in health facilities	Acceptable if<br>AUC>0.60 or IF 0.75-1.25<br>(Suggest indicators warranting incorporation into population-level surveys should meet both criteria- individual and population level validity)
Blanc et al. 2016[34], McCarthy et al. 2016[35]	Quality of maternal and newborn health care during childbirth	Individual-level accuracy measured by AUC<br>- High: AUC>0.7<br>- Moderate: 0.6<AUC<0.7<br>- Low: AUC<0.6<br>Degree of bias measured by IF<br>- Low 0.75<IF<1.25<br>- Moderate 0.5<IF<1.5<br>- Large IF<0.5 | IF>1.5<br>High overall performance: high AUC + low IF<br><br>Reliability (decay in accuracy between baseline taken during exit interview and at 13-15 months post delivery) – Phi coefficient (r_{phi})<br>- Poor if <0.4, Moderate between 0.4 and 0.6; High 0.6-0.8; >=0.8 almost perfect agreement.
Blanc et al. 2016[48]	Skilled birth attendant and key elements of maternal, intrapartum, newborn and immediate postnatal care among women with vaginal deliveries	Benchmarks of validity:<br>- AUC>=0.6<br>- 0.75<IF<1.25<br>Overall acceptable indicator performance: both AUC and IF benchmarks met
Munos et al. [41], Chang et al. 2018[49]	Coverage indicators	AUC≥0.70<br>IF 0.75 – 1.25<br>(study authors acknowledge these are arbitrary)

AUC: Area under the receiver operating characteristic curve; TAP: test to actual positive ratio; IF: inflation factor.

Convergent validity

The second common type of indicator validity compares estimates from various data sources or measurement approaches seeking to measure the same construct to understand the convergence between them. Studies assessing convergent validity aim to quantify the extent to which two or more estimates of a common construct, which should be related because they converge on the same

theoretical construct, are in fact related. Assessments of convergent validity are commonly used in situations where a "gold standard" does not exist or is infeasible to estimate. A typical question asked in assessments of convergent validity is the extent to which a new/different data source or estimation method compares to an established source or method. Studies also seek to understand the strengths and limitations, including financial feasibility, of the measurement approaches being compared. A wide range of methods has been used to examine the extent of agreement between distinct measurement methods and data sources used to calculate an indicator, including whether the data is on an individual, cluster (e.g. region, facility), or population level. Similarly to criterion validity, the cut-off point for an acceptable level of convergent validity is also subjective. Examples of studies assessing convergent validity are shown in Table 4.

Tab 4. Examples of approaches to assess convergent validity

Indicator	Estimation method 1	Estimation method 2	Comparison
Stillbirth rate, neonatal mortality rate[50]	Full history of all live births and questions on pregnancies in the last five years resulting in non-live births	Full history of all pregnancies and their outcomes	Crude and adjusted risk ratios (determinants and clustering)
Maternal and perinatal mortality[51]	Enhanced community-level surveillance system	Routine data	Comparison of rate/ratios
Postnatal care coverage[52]	DHS questions	MICS questions	Descriptive comparison of proportions and timing
Caesarean section rate[53]	Population-based survey (DHS, MICS)	Health facility records	Linear regression coefficient; confidence interval overlap
Antenatal care coverage and content[54]	Individual-level data from clinical records weighted to population level	Aggregate routine health information systems reports	Simple comparisons of proportions and 95% confidence intervals
Estimates of value of aid for RMNCAH[55]	Comparison of estimates from four initiatives: Countdown to 2015, the Institute for Health Metrics and Evaluation, the Muskoka Initiative, and the Organisation for Economic Co-operation and Development (OECD)		Simple differences

DHS - Demographic Health Surveys, MICS - Multiple Indicator Cluster Surveys, RMNCAH – Reproductive, maternal, newborn, child and adolescent health.

Construct validity

One of the most important types of indicator validity highlighted in key informant interviews was construct validity. An indicator provides a simplified way of capturing a more complex phenomenon. Construct validity can be defined as the accuracy of the operationalisation of such phenomenon. In other words, the question is not how valid an indicator is, but how valid is this specific measurement of

an indicator, in this place, at this time. Arnold and Khan call this process of transforming concepts into indicators and further into survey questions the “validity of question”.[56] The purpose of an indicator is central to assessing construct as well as other types of validity.[57] The importance of clearly understanding and articulating an indicator’s purpose was highlighted in a recent paper by Radovich and colleagues that examined the indicator of skilled birth attendant (SBA).[58] Several respondents emphasized that the process of assessing whether an indicator is “valid” should start with an understanding of not only the construct or phenomenon an indicator intends to measure, but also for whom and why. This includes a consideration of whether the underlying phenomenon itself is meaningful, that is, whether its purpose is important to maternal and newborn health and clearly understood by all stakeholders (S1 Table). Yet, despite the importance attributed to construct validity by key informants, there was comparatively less published literature within maternal and newborn health focusing on this topic.

Some of the key indicators currently in use in the field of maternal and newborn health were developed, or are being used, as proxies for constructs that are considered important by stakeholders but are not feasible or possible to measure simply or directly. This relates to, for example, maternal mortality (large sample sizes required means measurement is expensive)[59-61] and quality of care (a multi-dimensional construct requiring data on technical and clinical levels as well as patient’s experience).[62] In particular, recent work around indicators of care content and quality is concerned with the extent to which measurement methods can capture complex, multifaceted constructs. Examples of this type of validity research include indicators of quality of care from a woman’s perspective,[63] from a health facility perspective[64] indicators of complex care processes (e.g. case management of pre-eclampsia), indicators of autonomy and respectful care [65] and person-centered maternity care.[66] While this work forms a large part of the current validation research of maternal and newborn health indicators, it is not yet fully formed.

Discussion

Using mixed methods, we identified three common types of indicator validity used in the field of maternal and newborn health, all of which have a role in evaluating the performance of indicators. Key informant interviews revealed that a variety of definitions and interpretations of indicator validity exist, highlighting

the need to establish a common language and understanding of indicator validity among global and local maternal and newborn health stakeholders. We have attempted to synthesize key concepts and to present a typology of indicator validity that characterizes the varied ways in which the concept of validity is understood and assessed in the literature, indicator guidance documents and by a sample of maternal and newborn health stakeholders. We suggest that those who develop, assess or recommend maternal and newborn health indicators clarify their understanding of the various types of validity of studied or recommended indicators.

Despite the importance of construct validity highlighted in key informants' responses, we identified a gap in the literature and indicator guidance documents in explicitly describing and evaluating the underlying phenomena which various maternal and newborn health indicators seek to measure, and an absence of studies of construct validity in general. For example, is the SBA indicator intended to measure an enabling childbirth health care environment, coverage of good quality childbirth care, minimum safety levels during childbirth, or to be a proxy for maternal mortality? Conceptual understanding of the underlying phenomena that specific indicators are intended to measure may vary across stakeholders using the indicators and may change over time, but are rarely made explicit. There is a predominance of validation studies on the narrowest conceptualisation of validity – criterion validity – but the larger issue of the construct and its meaning for progress in maternal and newborn health is rarely addressed. Once developed, used and measured with a high uptake for many years, maternal and newborn health indicators tend to remain in use for decades. However, the constructs being measured by such indicators are often unclear or may evolve in importance over time. We also highlight a view shared by many key informants that an indicator's performance on assessment of criterion validity should not be the sole determinant of its use for monitoring and decision-making; its measurement parameters need to be "good enough" for the purpose at a given time and place.[23] One such aim could include generating aspirational indicator estimates for the purpose of improving quality of data or measurement methods for the future.[8]

There is a growing concern with the large number of maternal and newborn health indicators used across several initiatives, including the variation in indicator definitions and the resources required to produce such indicators.[6] A more consistent understanding of indicator validity could help guide the prioritization, development and testing of more robust maternal and newborn health indicators.[14]

Improved global coordination among stakeholders conducting or supporting validation studies is needed to avoid duplication of efforts. Further, it is crucial to consider the perspectives of country-level stakeholders in prioritising which types of validation matter most for which indicators and which types of indicators should be validated first and where. The development of guidance and criteria for assessing common types of indicator validity, linked to an action plan to prioritize indicators for validation, could help improve such coordination. Coordinated research to assess validity of a smaller number of locally relevant core indicators that seek to measure important constructs could help accelerate action to improve maternal and newborn health. In parallel, it is also vital to coordinate assessment of indicator validity with assessment of other important attributes of indicators, including feasibility and reliability.[67] Studies which describe elements of clarity, feasibility and acceptability of data collection tools,[68-71] such as those employed in qualitative studies and cognitive interviewing,[68] are complementary to other assessments of validity.

Limitations

This paper used a literature review and key informant interviews to explore the field of indicator validation research in maternal and newborn health indicators. We conducted a comprehensive review of the literature published in English since 1990 to identify key themes and provide examples and acknowledge that our review may have missed relevant publications in languages other than English. We also acknowledge that while the key informants included measurement experts and authors of many of the recently conducted validation studies on maternal and newborn health indicators within the maternal and newborn health field, our sample of key informants included only English-speaking respondents working predominantly at the global level and did not include many country-level experts and stakeholders. We did not aim to summarize the findings of all validation studies for individual indicators; however, such systematic reviews and meta-analyses could be a useful next step for summarising the available evidence.

Conclusion

Indicator validation is a part of a continuous process of building and synthesising evidence on indicator performance. We found that in the maternal and newborn health literature and among measurement experts, the term validity is used broadly to capture a variety of indicator performance assessments. Some of the current challenges related to harmonization and coordination of maternal and newborn

health indicators stem from a heterogeneity of definitions of indicator validity, often by stakeholders from various disciplinary backgrounds. We recommend that the language used to describe validation research should be more precise as to the specific type(s) of validation assessed and the related findings (e.g. an indicator described as “valid” or “validated” should be nuanced and time- and context-specific).

In addition to the three most common types of maternal and newborn health indicator validity identified, we highlight the fact that any appraisal of an indicator’s validity requires clarity about the construct that the indicator is intending to measure. We therefore recommend that future initiatives to coordinate indicator validity research focus on important underlying constructs rather than individual indicators (which represent the operationalization of constructs). This approach can help align stakeholders to develop a clear understanding of how best to measure important constructs, including agreement on “how not to measure” a construct for which “valid” indicators may not yet have been developed and tested.

Acknowledgments

The authors would like to acknowledge the key respondents' participation in interviews and discussions with members of the MoNITOR technical advisory group.

Disclaimer

This report contains the collective views of the authors and does not necessarily represent the decisions or the stated policy of the World Health Organization.

References

[1] Trends in maternal mortality 2000 to 2017: estimates by WHO, UNICEF, UNFPA, World Bank Group and the United Nations Population Division. Geneva: World Health Organization; 2019.

[2] United Nations Inter-agency Group for Child Mortality Estimation (UN IGME). Levels & Trends in Child Mortality Estimates developed by the UN Inter-agency Group for Child Mortality Estimation. New York: United Nations Children's Fund; 2018.

[3] Blencowe H, Cousens S, Jassir FB, Say L, Chou D, Mathers C, et al. National, regional, and worldwide estimates of stillbirth rates in 2015, with trends from 2000: a systematic analysis. Lancet Glob Health 2016;4:e98-e108.

[4] World Health Organization. Global Strategy for Women's, Children's and Adolescents Health (2016-2030). Geneva: WHO; 2015.

[5] United Nations. Sustainable Development Goals (http://www.un.org/sustainabledevelopment/sustainable-development-goals/ , accessed 14 Nov 2017).

[6] Moller AB, Newby H, Hanson C, Morgan A, El Arifeen S, Chou D, et al. Measures matter: A scoping review of maternal and newborn indicators. PLoS One 2018;13:e0204763.

[7] Larson C, Mercer A. Global health indicators: an overview. CMAJ : Canadian Medical Association Journal 2004;171:1199-200.

[8] Grove J, Claeson M, Bryce J, Amouzou A, Boerma T, Waiswa P, et al. Maternal, newborn, and child health and the Sustainable Development Goals--a call for sustained and improved measurement. Lancet 2015;386:1511-4.

[9] Munos MK, Stanton CK, Bryce J. Improving coverage measurement for reproductive, maternal, neonatal and child health: gaps and opportunities. J Glob Health 2017;7:010801.

[10] Carvajal-Aguirre L, Vaz LM, Singh K, Sitrin D, Moran AC, Khan SM, et al. Measuring coverage of essential maternal and newborn care interventions: An unfinished agenda. J Glob Health 2017;7:020101.

[11] Saturno-Hernandez PJ, Martinez-Nicolas I, Moreno-Zegbe E, Fernandez-Elorriaga M, Poblano-Verastegui O. Indicators for monitoring maternal and neonatal quality care: a systematic review. BMC Pregnancy Childbirth 2019;19:25.

[12] https://collections.plos.org/measuring-coverage-in-mnch.

[13] http://www.jogh.org/col-coverage-measurement.htm.

[14] Marchant T, Bhutta ZA, Black R, Grove J, Kyobutungi C, Peterson S. Advancing measurement and monitoring of reproductive, maternal, newborn and child health and nutrition: global and country perspectives. BMJ Glob Health 2019;4:e001512.

[15] Moran AC, Moller AB, Chou D, Morgan A, El Arifeen S, Hanson C, et al. 'What gets measured gets managed': revisiting the indicators for maternal and newborn health programmes. Reprod Health 2018;15:19.

[16] WHO. https://www.who.int/data/maternal-newborn-child-adolescent/monitor (Accessed July 31, 2019). 2019.

[17] Every Woman Every Child. The Global Strategy for Women's, Children's, and Adolescent's Health (2016–2030): Survive, Thrive, Transform New York, NY, USA: United Nations; 2015.

[18] Every Newborn: an action plan to end preventable deaths. . Geneva: World Health Organization; 2014.

[19] Strategies towards ending preventable maternal mortality (EPMM). Geneva: World Health Organization.

[20] Miles M, Huberman A. Qualitative Data Analysis: An Expanded Source Book. Sage, Newbury Park, CA. Cited in Jabareen Y (2009) Building a conceptual framework: philosophy, definitions and procedures. International Journal for Qualitative Methods 2008. 1994.

[21] Levac D, Colquhoun H, O'Brien K. Scoping studies: advancing the methodology. Implementation science 2010;5.

[22] Bragge P, Clavisi O, Turner T, Tavender E, Collie A, Gruen RL. The Global Evidence Mapping Initiative: scoping research in broad topic areas. BMC Med Res Methodol 2011;11:92.

[23] Benova L, Moller AB, Moran AC. "What gets measured better gets done better": The landscape of validation of global maternal and newborn health indicators through key informant interviews. PLoS One 2019;[to be updated once published].

[24] WHO. 2018 Global Reference List of 100 Core Health Indicators (plus health-related SDGs). Geneva: World Health Organization; 2018.

[25] Stevens GA, Alkema L, Black RE, Boerma JT, Collins GS, Ezzati M, et al. Guidelines for Accurate and Transparent Health Estimates Reporting: the GATHER statement. Lancet 2016;388:e19-e23.
[26] Sechrest L. Validity of Measures Is No Simple Matter. Health Research and Educational Trust 2005;40:1584-604.
[27] Messick S. Validity of Psychological Assessment: Validation of Inferences from Persons' Responses and Performances as Scientific Inquiry into Score Meaning. American Psychologist 1995;50.
[28] Streiner DL, Norman GR. “Precision” and “Accuracy”: Two Terms That Are Neither. Journal of Clinical Epidemiology 2006;59:327-30.
[29] Ronsmans C, Achadi E, Cohen S, Zazri A. Women's recall of obstetric complications in south Kalimantan, Indonesia. Stud Fam Plann 1997;28:203-14.
[30] Sloan NL, Amoaful E, Arthur P, Winikoff B, Adjei S. Validity of women's self-reported obstetric complications in rural Ghana. J Health Popul Nutr 2001;19:45-51.
[31] Souza JP, Cecatti JG, Pacagnella RC, Giavarotti TM, Parpinelli MA, Camargo RS, et al. Development and validation of a questionnaire to identify severe maternal morbidity in epidemiological surveys. Reprod Health 2010;7:16.
[32] Seoane G, Castrillo M, O'Rourke K. A validatiton study of maternal self reports of obstetrical complications: implications for health surveys. International Journal of Gynecology and Obstetrics 1998;62:229-36.
[33] Zurayk H, Khattab H, Younis N, Kamal O, el-Helw M. Comparing women's reports with medical diagnoses of reproductive morbidity conditions in rural Egypt. Stud Fam Plann 1995;26:14-21.
[34] Blanc AK, Warren C, McCarthy KJ, Kimani J, Ndwiga C, RamaRao S. Assessing the validity of indicators of the quality of maternal and newborn health care in Kenya. J Glob Health 2016;6:010405.
[35] McCarthy KJ, Blanc AK, Warren CE, Kimani J, Mdawida B, Ndwidga C. Can surveys of women accurately track indicators of maternal and newborn care? A validity and reliability study in Kenya. J Glob Health 2016;6:020502.
[36] Stanton CK, Rawlins B, Drake M, Dos Anjos M, Cantor D, Chongo L, et al. Measuring coverage in MNCH: testing the validity of women's self-report of key maternal and newborn health interventions during the peripartum period in Mozambique. PLoS One 2013;8:e60694.
[37] Liu L, Li M, Yang L, Ju L, Tan B, Walker N, et al. Measuring coverage in MNCH: a validation study linking population survey derived coverage to maternal, newborn, and child health care records in rural China. PLoS One 2013;8:e60762.
[38] Day L-T, Ruysen H, Gordeev V, al e. “Every Newborn-BIRTH” study protocol: Observational Study Validating indicators for coverage and quality of maternal and newborn health care in Bangladesh, Nepal and Tanzania. Journal of Global Health 2019;(to be updated once published).
[39] Demographic and Health Survey (accessed April 4, 2019) https://dhsprogram.com/What-We-Do/Survey-Types/DHS.cfm.
[40] Multiple Indicator Cluster Surveys (accessed April 4, 2019 http://mics.unicef.org/).
[41] Munos MK, Blanc AK, Carter ED, Eisele TP, Gesuale S, Katz J, et al. Validation studies for population-based intervention coverage indicators: design, analysis, and interpretation. J Glob Health 2018;8:020804.
[42] Ronsmans C. Studies validating women's reports of reproductive ill health: How useful are they? Seninar Innovative approaches to the assessment of reproductive health (IUSSP). Manila, the Philippines1996.
[43] Vecchio T. Predictive value of a single diagnostic test in unselected populations. New England Journal of Medicine 1966;274:1171-3.
[44] Betran AP, Torloni MR, Zhang JJ, Gulmezoglu AM. WHO Statement on Caesarean Section Rates. Bjog 2016;123:667-70.
[45] Souza JP, Betran AP, Dumont A, de Mucio B, Gibbs Pickens CM, Deneux-Tharaux C, et al. A global reference for caesarean section rates (C-Model): a multicountry cross-sectional study. Bjog 2016;123:427-36.
[46] Day LT, Ruysen H, Gordeev VS, Gore-Langton GR, Boggs D, Cousens S, et al. "Every Newborn-BIRTH" protocol: observational study validating indicators for coverage and quality of maternal and newborn health care in Bangladesh, Nepal and Tanzania. J Glob Health 2019;9:010902.
[47] Moran AC, Kerber K, Pfitzer A, Morrissey CS, Marsh DR, Oot DA, et al. Benchmarks to measure readiness to integrate and scale up newborn survival interventions. Health Policy Plan 2012;27 Suppl 3:iii29-39.
[48] Blanc AK, Diaz C, McCarthy KJ, Berdichevsky K. Measuring progress in maternal and newborn health care in Mexico: validating indicators of health system contact and quality of care. BMC Pregnancy Childbirth 2016;16:255.

[49] Chang KT, Mullany LC, Khatry SK, LeClerq SC, Munos MK, Katz J. Validation of maternal reports for low birthweight and preterm birth indicators in rural Nepal. J Glob Health 2018;8:010604.
[50] Baschieri A, Gordeev V, et al. Every Newborn-INDEPTH” (EN-INDEPTH) study protocol for a randomised comparison of household survey modules for measuring stillbirths and neonatal deaths in five Health and Demographic Surveillance sites. Journal of Global Health 2019;9:doi: 10.7189/jogh.09.010901.
[51] Anwar J, Torvaldsen S, Sheikh M, Taylor R. Under-estimation of maternal and perinatal mortality revealed by an enhanced surveillance system: enumerating all births and deaths in Pakistan. BMC Public Health 2018;18:428.
[52] Amouzou A, Mehra V, Carvajal-Aguirre L, Khan SM, Sitrin D, Vaz LM. Measuring postnatal care contacts for mothers and newborns: An analysis of data from the MICS and DHS surveys. J Glob Health 2017;7:020502.
[53] Stanton CK, Dubourg D, De Brouwere V, Pujades M, Ronsmans C. Reliability of data on caesarean sections in developing countries. Bull World Health Organ 2005;83:449-55.
[54] Venkateswaran M, Mørkrid K, Abu Khader K, Awwad T, Friberg IK, Ghanem B, et al. Comparing individual-level clinical data from antenatal records with routine health information systems indicators for antenatal care in the West Bank: A cross-sectional study. PLOS ONE 2018;13:e0207813.
[55] Pitt C, Grollman C, Martinez-Alvarez M, Arregoces L, Borghi J. Tracking aid for global health goals: a systematic comparison of four approaches applied to reproductive, maternal, newborn, and child health. Lancet Glob Health 2018;6:e859-e74.
[56] Arnold F, Khan SM. Perspectives and implications of the Improving Coverage Measurement Core Group's validation studies for household surveys. J Glob Health 2018;8:010606.
[57] Fischer C, Anema HA, Klazinga NS. The validity of indicators for assessing quality of care: a review of the European literature on hospital readmission rate. Eur J Public Health 2012;22:484-91.
[58] Radovich E, Benova L, Penn-Kekana L, Wong K, Campbell OMR. 'Who assisted with the delivery of (NAME)?' Issues in estimating skilled birth attendant coverage through population-based surveys and implications for improving global tracking. BMJ Glob Health 2019;4:e001367.
[59] Storeng KT, Behague DP. "Guilty until proven innocent": the contested use of maternal mortality indicators in global health. Crit Public Health 2017;27:163-76.
[60] Graham WJ, Campbell OM. Maternal health and the measurement trap. Soc Sci Med 1992;35:967-77.
[61] Graham WJ, Ahmed S, Stanton C, Abou-Zahr C, Campbell OM. Measuring maternal mortality: an overview of opportunities and options for developing countries. BMC Med 2008;6:12.
[62] Tuncalp, Were WM, MacLennan C, Oladapo OT, Gulmezoglu AM, Bahl R, et al. Quality of care for pregnant women and newborns-the WHO vision. Bjog 2015;122:1045-9.
[63] Tripathi V, Stanton C, Strobino D, Bartlett L. Development and Validation of an Index to Measure the Quality of Facility-Based Labor and Delivery Care Processes in Sub-Saharan Africa. PLoS One 2015;10:e0129491.
[64] Sheffel A, Karp C, Creanga AA. Use of Service Provision Assessments and Service Availability and Readiness Assessments for monitoring quality of maternal and newborn health services in low-income and middle-income countries. BMJ Glob Health 2018;3:e001011.
[65] Vedam S, Stoll K, McRae DN, Korchinski M, Velasquez R, Wang J, et al. Patient-led decision making: Measuring autonomy and respect in Canadian maternity care. Patient Educ Couns 2018.
[66] Afulani PA, Diamond-Smith N, Golub G, Sudhinaraset M. Development of a tool to measure person-centered maternity care in developing settings: validation in a rural and urban Kenyan population. Reproductive Health 2017;14:118.
[67] World Health Organization. Millennium Development Goals. The health indicators: Scope, definitions and measurement methods. Geneva: WHO; 2003.
[68] Hill Z, Okyere E, Wickenden M, Tawiah-Agyemang C. What can we learn about postnatal care in Ghana if we ask the right questions? A qualitative study. Glob Health Action 2015;8:28515.
[69] Chang KT, Mullany LC, Khatry SK, LeClerq SC, Munos MK, Katz J. Why some mothers overestimate birth size and length of pregnancy in rural Nepal. J Glob Health 2018;8:020801.
[70] Yoder PS, Rosato M, Mahmud R, Fort A, Rahman F, Armstrong A, et al. Women’s Recall of Delivery and Neonatal Care: A Study of Terms, Concepts, and Survey Questions. Calverton, Maryland, USA: ICF Macro; 2010.
[71] Hussein J, Hundley V, Bell J, Abbey M, Asare GQ, Graham W. How do women identify health professionals at birth in Ghana? Midwifery 2005;21:36-43.

Supporting information captions

S1 Table. Overview of key response themes from interviews with key informants

Fig1

Click here to access/download;Figure;Fig1.tiff

Indicator domain	Main data source(s)	Examples of indicators	Detail of an example indicator* (N-numerator, D-denominator)
1<br>Health system	Country-level informants (MoH, MoF, MoE)<br>Policy documents<br>Household expenditure surveys	• Newborn lifesaving commodities in essential medicine list<br>• Birth registration<br>• Costed national implementation plan for maternal, newborn and child health	**Newborn lifesaving commodities in essential medicine list**<br>Number of the four listed commodities that are included in the essential medicine list (injectable antibiotics, antenatal corticosteroids, chlorhexidine and resuscitation equipment).
2<br>Access to and availability of care	Facility surveys/ censuses<br>Facility records (stock cards)<br>HMIS	• Availability of bag and mask for newborn resuscitation<br>• Availability of functional EmONC facilities	**Availability of bag and mask for newborn resuscitation**<br>N: Number of facilities with a functional neonatal bag and two masks (sizes 0 and 1) in the labour and delivery service area<br>D: Total number of facilities with inpatient maternity services that were assessed
3<br>Care coverage	Population-level surveys<br>Facility records/ observations<br>HMIS	• Kangaroo mother care<br>• Newborn resuscitation<br>• Antenatal care (at least 4 visits)<br>• Institutional (facility-based) delivery	**Kangaroo mother care**<br>N: Number of newborns initiated on facility-based kangaroo mother care<br>D: Target population for coverage: total number of newborns with birthweight <2000g or <2500g
4<br>Care content and quality	Facility surveys/ censuses<br>Facility records/ observations<br>User surveys	• Antenatal care: blood pressure measured<br>• Person-centeredness of care<br>• Labor and Delivery Quality of Care Short Observational Index<br>• Respectful maternity care	**Antenatal care: blood pressure measured**<br>N: Number of women who attended antenatal care who had blood pressure measured<br>D: Number of women who attended antenatal care (denominator definition might vary depending on data source)
5<br>Impact	Population-level surveys<br>Civil registration vital statistics<br>Facility records<br>HMIS	• Maternal mortality ratio (per 100,000 live births)<br>• Stillbirth rate (per 1,000 total births)<br>• Neonatal mortality rate (per 1,000 live births)<br>• Percentage of women aged 20–24 years who gave birth before age 18	**Maternal mortality ratio (per 100,000 live births)**<br>N: Number of maternal deaths during a given time period<br>D: Live births during the same time period x 100,000

* Indicator definitions may vary by data source and indicator list used.

Notes: EmONC: Emergency obstetric and newborn care; HMIS: health management information system (i.e. aggregate, routinely collected health service data); MoH: Ministry of Health; MoF: Ministry of Finance; MoE: Ministry of Education.

Fig2

Click here to access/download;Figure;Fig2.tiff

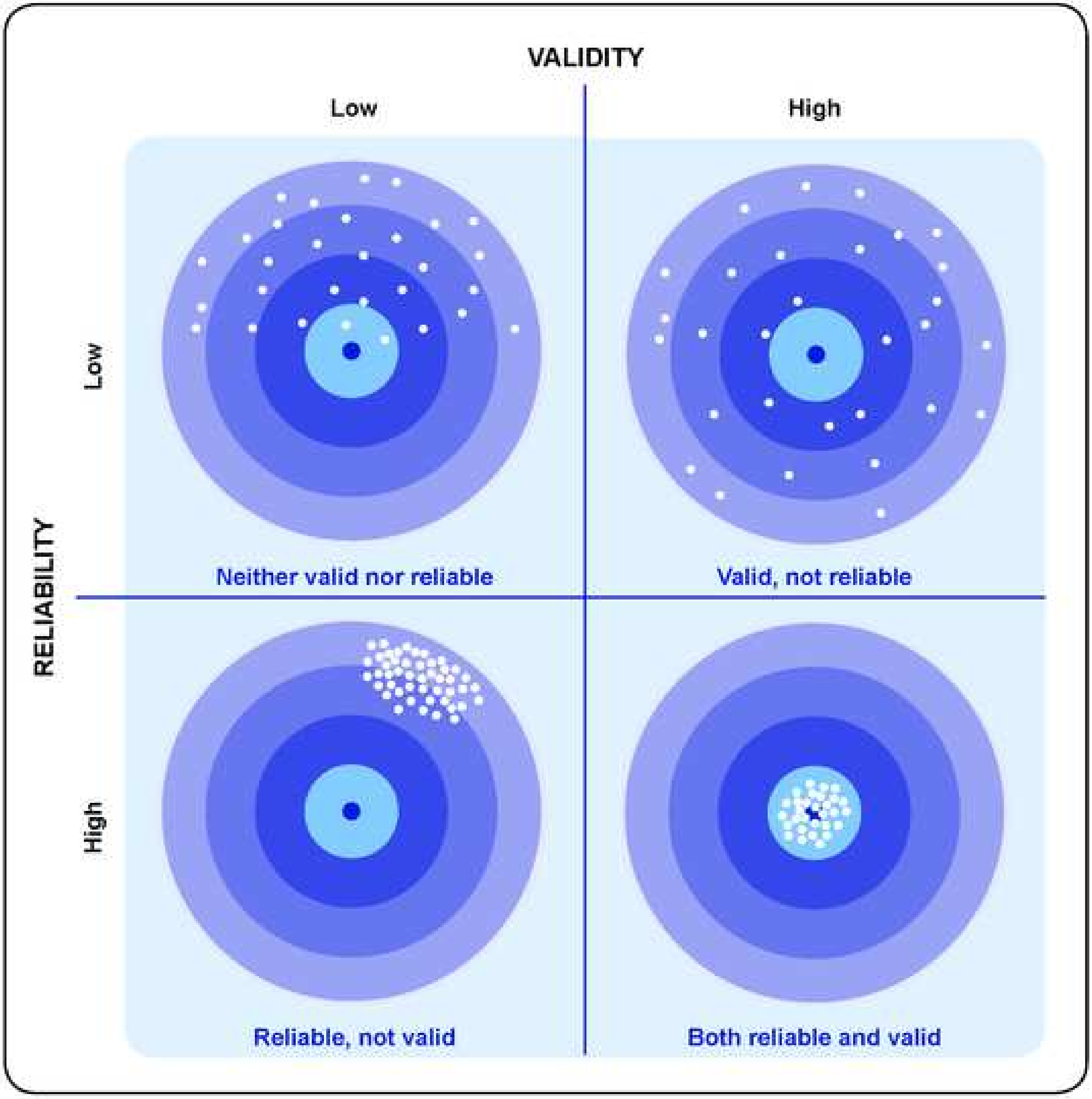

S1 Table

Click here to access/download
Supporting Information
S1 Table.docx
