# Supplementary material for: What is meant by validity in maternal and newborn health measurement? A conceptual framework for understanding indicator validation
Source: PLoS One. 2020 May 29;15(5):e0233969. doi: 10.1371/journal.pone.0233969 (PMC7259779; doi:10.1371/journal.pone.0233969)
Supplement: Attachment — Submitted filename: Comments to Editors Nov_2019.docx [file pone.0233969.s003.docx]

PLOS ONE

Manuscript Number: PONE-D-19-29431

Title: What is meant by validity in maternal and newborn health measurement? A conceptual

framework for understanding indicator validation

Short Title: Conceptual framework for validity of maternal and newborn health indicators

Article Type: Research Paper

Keywords: Maternal health; newborn health; Measurement; indicators; validation; validity

Comments to Editors:

Minor Essential Revisions: This is a very interesting article; however there are some aspects that need to be incorporated in some sections in order to improve the manuscript and comprehension, especially regarding methods.

Specific Comments:

Abstract: Question posed: the question posed is clear, it should include the purpose of the study.

Introduction: complete and updated, the objectives of the study are clearly stated

Methods:

Authors mentioned that this paper was developed iteratively, guided by principles for conceptual framework defined by Miles and Huberman (20), they should provide readers a brief description or definition about this framework and how this applies to this paper, not just mention. They also mention having used a purposive sampling to identify key informants and having finally interviewed 32 respondents from different backgrounds, it is important and interesting having the description of these different backgrounds.

Results:

I suggest authors to include a briefly description on finally how many studies were included in this review and a scheme showing the way they were selected and excluded.

In my opinion the heading Key terms in indicator validity line 174 page 6, containing definitions, should be moved to the introduction, or the methods section, this is not part of the results. The same occurs with the text written from line 223-227 in page 8, in my opinion authors discussed their findings, and therefore this should be moved to the discussion section. Some limitations are stated in this section lines 237-242, which in my opinion should be moved to the corresponding section.

Discussion:

Complete and comprehensive, see the above comment to be considered with regard the limitations of this study.

Conclusion: adequate.

Tables and figures:

I suggest to include a figure or scheme describing the number of studies included those selected and those excluded, this will help to clarify the reader´s comprehension.

Author’s contribution: Not mentioned

Ethics Statements: No ethics approval was required because of the nature of this study.

Conficts of interest: "The authors have declared that no competing interests exist."

References:

This section follows the journal requirements.
