# Supplementary material for: What is meant by validity in maternal and newborn health measurement? A conceptual framework for understanding indicator validation
Source: PLoS One. 2020 May 29;15(5):e0233969. doi: 10.1371/journal.pone.0233969 (PMC7259779; doi:10.1371/journal.pone.0233969)
Supplement: Attachment — Submitted filename: PLOS ONE indicators review 261119.docx [file pone.0233969.s004.docx]

**Reviewer’s Report 1: PONE-D-19-29431: What is meant by validity in maternal and newborn health measurement? A conceptual framework for understanding indicator validation.**

Thank you for the opportunity to review the manuscript.

The authors should be congratulated on a well-written and timely paper presenting a range of perspectives on how validity of maternal and newborn indicators is defined and understood by those who develop and use these indicators. The study has important implications for ongoing global efforts to track maternal and newborn health progress.

Please see below for specific comments on the manuscript:

Materials and methods

- Line 117: Please reference the key indicator guidance documents used in the review.
- Line 22: Can the authors please provide further details about the key informants’ backgrounds/expertise on this topic or in MNH? The authors provide some background information in the limitations section of the discussion however it would be useful to have this outlined earlier. Were all key informants’ part of the MoNITOR group?
- Was there any pre-testing of the interview guide prior to the 32 key informant interviews?
- What was the consent process?
- Reference 23: Is this paper accepted for publication or under review? Please update reference.
- Line 127: What written materials and relevant publications were received from the respondents after the interview? How were these relevant to the interviews?
- Can the authors please clarify the process for the qualitative data analysis? Apologies if I have overlooked this. Was the analysis undertaken by the same person who undertook the interviews?

Results

- Line 223: The authors state that key informants noted that a substantial portion of recent work on assessing criterion validity has focused on indicators of care coverage. It would be useful to know what proportion of the key informants identified this to be the case.

The discussion is well-written, and the conclusion is concise and logical. This is an interesting and useful paper that should be considered for publication following revision of comments.
