# Supplementary material for: What is meant by validity in maternal and newborn health measurement? A conceptual framework for understanding indicator validation
Source: PLoS One. 2020 May 29;15(5):e0233969. doi: 10.1371/journal.pone.0233969 (PMC7259779; doi:10.1371/journal.pone.0233969)
Supplement: Attachment — Submitted filename: 20200124_Response to reviewers.docx [file pone.0233969.s005.docx]

Dear Dr Salinas-Miranda	January 24, 2020

Thank you very much for considering our manuscript. Your and the reviewers’ suggestions on how to strengthen it are very appreciated. Below we respond to the feedback point-by-point.

| **Comment** | **Response** |
| --- | --- |
| **Academic editor and journal requirements** |  |
| The paper is well-written and has adequate scientific value. The paper has been reviewed by independent reviewers who agree with the value of the manuscript for the readership of PLOS ONE and the important contribution to the literature. Hence, the recommendation is to accept upon minor revisions. Given the comprehensive nature of the manuscript that required diverse expertise, the Academic Editor requested more than the minumum number of reviewers and invited reviewers with different expertise (in maternal and child health, measurement, and epidemiology). The reviewers generally agreed with the need to make some changes in the manuscript, particularly related to the definition of validity and measurement validity. Please make the indicated corrections and resend a revised version. | Thank you for your comments and questions. We address these below in our responses to the reviewers. |
| There was a general agreement among reviewers about the need to improve the explanations of the different types of validity discussed. | We have addressed the reviewers’ comments by:  Improving the definitions of validity as applicable to the field of health indicators in general (in the Introduction section), and maternal/newborn indicators in particular (in the Results section, as these are findings based on our literature review and key informant interviews). We have included a new Figure (Figure 3) to help explain the three types of validity.  We also added text to acknowledge (briefly) the differences in the definitions we use and definitions used by other fields (social sciences, psychology, etc). This will also help explain why we do not mention (at all or extensively) some types of validity, such as face, content validity etc. |
| We ask that you please provide a bit more detail in you methods section about the interviews and framework:  Thank you for providing a reference (20) for the guiding principles used in this study. We request that you please provide a brief description as well as a citation. If materials, methods, and protocols are well established, authors may cite articles where those protocols are described in detail, but the submission should include sufficient information to be understood independent of these references ([https://journals.plos.org/plosone/s/submission-guidelines#loc-materials-and-methods](https://protect-eu.mimecast.com/s/iP4zCmy54u1mnzHBsa3H?domain=journals.plos.org)).  Additionally, we ask that you please provide a bit more information about the recruitment of key informants, how they were identified and invited to participate in the study, as well as a description of the questions they were asked during the interview. This can be added as Supporting Information or in the methods section. | We have expanded the description of the conceptual framework (Reviewer 4), and provided more information about the methods used in the literature review and key informant interviews (a separate paper has now been published, and is referenced in full). |
| **Reviewer 1** |  |
| The topic covered by this paper is well timed, and well needed. How validity is defined, and ultimately what should be considered a valid maternal and newborn health indicator is important. As such, the paper has potential to provide much needed guidance in the field, and help to focus the 140+ MNCH indicators currently in play. This paper has immense potential. I hope these observations are useful. | We thank the reviewer for their time and suggestions, which have improved the quality of this manuscript. |
| However, the authors would benefit from setting the stage early of what is meant by validity, and if they intend to also talk about reliability. This would mean a thorough (though concise) review of the definitions of validity, anchored with multiple strong referrences published in peer reviewed journals or text books. This will help readers also know what to look for when reading the author's results, and may help the authors taylor the way they present their results, key points they highlight, and what they ultimately discuss. There are a couple of types of validity - namely face validty, and content validity, both important, that are missing.  The authors did a great job reviewing teh MNCH literature, but they are also encouraged to review the validity literature, align their definitions of the various types of validity with those commonly understood within the literature, and then review their data and update their analysis in light of these revised definitions of validity. | Thank you for your constructive feedback and comments. We appreciate these detailed suggestions and links to further reading. We have incorporated them in the manuscript.   1. We moved the definition of validity to the Methods section and expanded it. 2. We added text to acknowledge that various disciplines have differing perspectives on the meaning of “validity” (with supporting references) and that we focus on the issues relevant to health indicators in general, and maternal and newborn indicators in particular.   The reviewer mentions that we should align our definitions of the various types of validity to “those commonly understood within the literature”. However, the literature on validity, types of validity and their definition/measurement are not uniform across disciplines. We struggled with this issue within the co-author team – to what extent do we align with one discipline’s definitions of validity and how do we triangulate these with the types of validity commonly used in maternal and newborn indicator measurement? We had rich data (from the key informants and literature review) on the various methods used to assess validity, but these accounts varied in their use of disciplines and language to describe and define validity. In fact, we consider this one of the most important findings of this paper – and had attempted to improve the field of maternal and newborn health measurement by proposing a unified framework and definitions of validity. Hence, our proposed definitions and classifications might not fit perfectly into those of other fields and disciplines. One example of this is the relationship between validity and reliability, where we take the stand that it is possible for a measurement of an indicator to be unreliable and still broadly valid (Figure 2). In the maternal and newborn health indicator field, this might happen with measures that have suboptimal sensitivity and/or specificity levels, yet the inflation factor performs satisfactorily (we have added this to the manuscript).  To address your comment about certain types of validity (face validity, and content validity) being absent from the framework, we expanded the text related to quality of care indicators.  Content validity in the psychometric literature refers to sampling from all types of items from which a construct is composed. Assessing content validity involves examining the degree to which the content of the test matches the whole content domain associated with the construct. It is not particularly relevant to the field of maternal and newborn indicators as one indicator (for example, % of births with a skilled birth attendant) is not supposed to or expected to measure many various dimensions of a complex construct. It is more relevant to the issues of measuring, quality of care, respectful care, or patient-centered care. We have added this to the text related to construct validity. |
| The definitions of criterion, construct, and convergence validity are also wrong or incomplete. Here are a couple of sources quickly found online: [https://socialresearchmethods.net/kb/convdisc.php](https://protect-eu.mimecast.com/s/FWC9CyXjgi6lMQTPaK6L?domain=socialresearchmethods.net) - helpful for all validity discussions re def) [https://www.scribbr.com/methodology/types-of-validity/](https://protect-eu.mimecast.com/s/OY0RCzXkji802yiM9DZo?domain=scribbr.com) | We thank the reviewer for this comment. We have expanded the definitions of these types of validity (as identified in the field of maternal and newborn health) and reference relevant literature.  We have also added a new Figure (Figure 3) to help explain exactly what we mean by criterion, convergent, and construct validity in the field of maternal and newborn health indicator measurement. |
| In addition, the authors could explicitly state whether or not some indicators can be judged as "more valid" than others if they meet more than one validity criteria. Ideally, a few examples could be provided of indicators and which criteria they meet. Until the definitons of validity are placed well, the authors will find it difficult to concretely discuss the range of how validity is defined within MNCH, and (hopeffully) highlight key gaps if they exist. | We thank the reviewer for raising this question, which we debated extensively in the co-author team. One of the main findings of this portfolio of work is that this is very much a grey area. From the key informant interviews (extensively reported in our other [paper](https://journals.plos.org/plosone/article?id=10.1371%2Fjournal.pone.0224746)), we found that the level of sensitivity/specificity etc which can be regarded as acceptable very much depends on the intended use of an indicator, the context, data sources, etc. Indicators need to be assessed in terms of whether they relate to the construct of interest, from a source that is consistent, or can be made so. There is a lot more work needed on the nuances of validity and reliability where indicators, versus other forms of measurement, are concerned.  We strongly believe that it is not within the scope of this manuscript describing the development of a conceptual framework to prescribe and justify “acceptable” levels of validity for the broad range of indicators currently in use in differing global settings. Such work is ongoing at the moment, within the portfolio of the MoNITOR group, and will result in a guidance document for validation studies. |
| The authors also need to clarify the relationship between reliability and validity. While it is true that an indicator can be reliable without being valid, this is not a reciprical relationship: an indicator cannot be valid wtihout being reliable. Please see here: [https://wonderlic.com/validity-and-reliability/](https://protect-eu.mimecast.com/s/vtdECA1pkcrvWPCB9Q0Y?domain=wonderlic.com) | We thank the reviewer for highlighting this issue and helping us clarify our statement on this. We have also debated this situation among the team. It is important to acknowledge that theoretically an indicator can be valid but unreliable (as per the bullseye illustration), despite this being counterintuitive and uncommon outside quantitative disciplines. |
| Overall on the analysis, since all data was qualitative, it would be useful to highlight key points with an occasional quote, if possible, or demonstrate a sense of whether or not certain opinions are those of only one person or multiple people. Key informants do not seem to contribute much to the discussion of criterion or convergent validity, though it would strenghten the study to integrate a bit more from Key informants on these topics. | We thank the reviewer for this suggestion. We have published a separate paper with the findings of the key informants’ interviews. In the original submission for this conceptual paper, we hesitated to duplicate the findings, and the key points are presented in a Supplementary Material (S1 Table). Based on your feedback, we have added more information about the perspectives of the key informants in the Results, including several quotes. |
| **Comments from marked up PDF document –from Reviewer 1** |  |
| Key terms in indicator validity:  Consider placing this secton earlier to set the stage, and then present the finding that it falls within the three definitions. | Thank you. We placed the overall definition of validity in the Methods, and retained the three sub-types of validity identified in the field of maternal and newborn health indicators in the Results section. |
| Key terms in indicator validity, referring to statement that assessment of validity should be done in conjunction with assessment of reliability and also potentailly feasibility.  “This should come sooner, and perhaps should be reflected in the title as well, since you are clearly not only interested in validity but also reliability” | Thank you for this suggestion – we moved the paragraph on Reliability from the Results section to the Definitions section in Methods. Given the strong focus of this work on validity (including the key informant interviews), we prefer to only keep the concept of “validity” in the title. We will add the word “reliability” to the key words of the article, however. |
| Criterion validity definition (line 184)  The authors could better describe criterion validity here and bring out the idea that it is actually measuring the extent to which a measure is related to an outcome. This would allow them to also talk about the concept of predictive validity which is increasingly becoming important in public health and could make a difference in maternal and newborn health as well... | Thank you for this observation; we have expanded the definition in the text and added Figure 3. |
| Convergent validity  A better, more indepth definition of convergent validity could help here, especially also with a mention of its opposite - - discriminate validity. I think its' about two constructs being similar, and not two measures measuring the same construct... (see https://socialresearchmethods.net/kb/convdisc.php - helpful for all validity discussions re def) | Thank you, and we address this in our revision. Your comment has helped us explain better that there are actually two separate types of convergent validity, which we now show in the example in Figure 3. |
| Construct validity  A better definition of construct validity is needed here that does not use the word construct in its definition... | Thank you, we have revised the wording. |
| First sentence in paragraph on Convergent validity (line 274-5) (“The second common type of indicator validity compares estimates from various data sources or measurement approaches seeking to measure the same construct to understand…” this refers to criterion validity, see ref: https://www.scribbr.com/methodology/types-of-validity/ | Thank you and we highlight here that the key difference is that with convergent validity, as we present in this manuscript, neither of the measures or indicators is a gold standard. |
| Table 4  Consider similar layout as Tab 3 that includes source | Thank you for this suggestion. We provide references (number) to the sources in the first column and would prefer that this table’s focus remains on the comparisons, rather than adding another column (or more text) listing the first author. |
| **Reviewer 2** |  |
| The paper presents an important perspective on how validity of maternal and newborn indicators can be defined and understood. This is important especially considering that the definitions and data sources used to produce indicator estimates vary and challenges exist with completeness, accuracy, transparency, and timeliness of data in many LMICs. Generally, the paper is well written and a few points to consider are as below. | We thank the reviewer for a careful reading of the manuscript. |
| Introduction: - Well summarized including the global and regional burden of the problem highlighting LMICs. - References used are up-to-date | Thank you. |
| Materials and Methods: - Overall good structure followed | Thank you. |
| Interviews were conducted by one interviewer between December 2017 and November 2018. Were the interviews conducted face-to-face, by telephone or skype?  One interviewer conducted the data collection from the 32 participants over a period of time. Can the authors indicate why recording the interviews was not opted for, yet it is an easier and faster way of collecting qualitative interviews and minimizes recall bias from the interviewer perspective? In addition, it would be nice for the authors to summarize the backgrounds of the participants interviewed.  Only one interviewer did the conducted and performed the shorthand note taking between 45 – 90 minutes. How was this structured?  How was the data from the 32 interviewees analysed? There is no analysis plan in the manuscript. | We thank the reviewer for these suggestions. Please note we have published a separate paper with details of the key informant interview findings. We have added information on the elements requested by the reviewer in the Methods section – in brief – and refer to the other paper (open access) where necessary. |
| Ethics – data collected involved interviewing human subjects on the subject matter. Why was ethical approval not sought by the authors? | No ethics approval was required. Key informant interviews were conducted about indicators and not human data or human tissue, we did not require an ethics approval.  The data in the form of interview notes is available open access under the DOI:  <https://doi.org/10.17037/DATA.00001403>  All key informants were asked to review the interview notes and agreed to have their anonymised interview notes included in this open access data file.  A separate paper was published on the key informant interviews, it is available here:  <https://journals.plos.org/plosone/article?id=10.1371%2Fjournal.pone.0224746> |
| Results and Discussion  Authors have reported the results in a good style with sub-headings separating the various aspects. | Thank you. |
| It is important that the authors highlighted the issue of subjectivity in the acceptable levels of validity in the criterion and convergent validity. | Thank you. |
| Main points have been well summarized in the discussion section | Thank you. |
| References - Robust review of literature done | Thank you. |
| Authors to review references 1,2,4,17,19 and 24 to include the URLs/web links and access dates as these are online/electronic sources as per the recommended citation style. | Thank you for a detailed review.  References 1,2,4,17 and 24 are reports, we do not believe that URLs are required according to the journal style, but are happy to do so given further guidance from the Editor.  We have completed reference 19 according to Journal citation style guidance. |
| **Reviewer 3** |  |
| Reviewer #3: The topic addressed is an important area in which some confusion is common, in general, ie not limited to maternal health. So, this manuscript has potential to make a useful contribution to help clarify issues within this field. However, there are two aspects which I think need improvement to strengthen this work. | We thank the reviewer for their time and expertise. |
| i) the three types of validity discussed belong within an established theoretical framework. I recommend that this be acknowledged more explicitly, by providing the definitions of these in the introduction rather than in the results, perhaps with a little more reference to the diversity of types of validity considered in literature. | We thank the reviewer for this suggestion. In light of other comments from the six reviewers and the Editor, we have opted to include the general definition of validity and reliability in the Methods section. The three specific types of validity assessment found in the maternal and newborn health measurement field are our findings, and we have opted to retain these in the Results section.  We acknowledge that there are many other types of validity, which are not used in or relevant to the field of maternal and newborn health indicators, and we chose not to mention these in great detail in this manuscript. |
| ii) The methods indicate that a literature review was conducted (line 117). Although the methods for identification of literature are stated (lines 131-8) the results provide very limited information regarding the literature identified, not even the number of manuscripts identified is mentioned. It is for this reason that I have indicated that all data underlying finding are not made fully available. More details on the findings should be provided. | We thank the reviewer for this comment, which has been addressed in the Methods section. |
| Lines 341-3 - several potential uses of the indicator are mentioned. It is however unclear why the discussion seeks to align the indicator with only one use, when in fact it may fulfil each of the stated uses in differing contexts. | We thank the reviewer for spotting this. We did not intend to suggest each indicator can only link to one underlying phenomenon, and we have rephrased this sentence to reflect this. |
| Lines 353-5 - the intended meaning is unclear. | Thank you for bringing this to our attention. We have now expanded the text in the Results section about acceptable levels of validity; this is also extensively described in our other paper (key informant interviews, to which the link points). We believe in light of these amendments this statement now makes sense. |
| Line 479 - journal details for ref [26] need to be corrected. | Thank you for the detailed review. We have corrected the journal name. |
| There are a number of occurrences of the term ‘this paper’, some of which could be dropped. | Thank you for noticing; we have gone through the manuscript and reduced the use of this phrase (now only appears three times, down from eight). |
| Other minor matters for attention: Line 169 - refers to S2 Table, but I could not locate it | Thank you for letting us know, we have corrected this typo which should have referred to S1 Table. |
| Line 186 – surely multiple measurements need to be compared with the relevant ‘gold standard’ measurements to examine criterion validity. | We mean here one occasion for measurement (for example, one survey), where indeed many people or facilities are sampled and their measurements taken, but from which one indicator estimate is derived. Each individual observation and gold standard will be used to calculate measures of criterion validity (sensitivity, specificity, etc), which again are summary measures of the differences. |
| Lines 192-3 - please clarify how the statement is related to the concept of convergent validity. | We have rephrased this statement. |
| Lines 247-50 - examples are useful but please provide a methodological reference for lines 247-8. | We based this statement on the results of key informant interviews, and have expanded this. |
| Lines 223-5 - this statement does not appear to be supported within the examples provided in Table 1. | Table 1 includes a range of criterion validity assessments, including fairly old studies (such as many of those in the “Impact” row. We do list a number of validation studies linked to the performance of questions/indicators derived from population-level studies, such as DHS and MICS, in the rows looking at indicators of Care coverage and Care content/quality. |
| Line 303 - more detail of the indicator needs to be provided: birth attended by ? Are lines 303-4 referring to source 58 or to the interviewees of the reported study? | Thank you for highlighting this, we have provided a definition of this indicator. |
| Tables and Figures I found it confusing that text refers to Tables and Figures, but they are entitled Tabs and Figs | Thank you, we have corrected this. |
| It is unclear what is plotted in each sub-plot in Figure 2 – what do the two axes represent? In line 206 reference to hitting the bullseye is confusing since the plot for high validity and low reliability has points scattered around the bullseye but spread across the entire circular region. | Thank you for this question. We intended to provide an example of the relationship between validity and reliability, such as is often shown in the research methods literature (for example: <https://courses.lumenlearning.com/suny-hccc-research-methods/chapter/chapter-7-scale-reliability-and-validity/>). The axes are intentionally unlabelled so that they help demonstrate a generic measurement of any indicator.  We have added clarifications to the text describing this Figure in the Methods sections. |
| In table 1 the headings could be clearer, eg in the second column the content is: “Indicator: gold standard / setting (study/ies”); since impact indicators are reported to be less common it is surprising that they appear first in the table. | Thank you for alerting us to this, we have renamed the first column of Table 1 “Indicator domain” to link to our definitions in the Methods section. We do state in the text just above Table 1 that impact indicators have been assessed using criterion validity. Given your comment, we have re-arranged the order of the rows in this table to follow the order of the five indicator dimensions as listed in the Methods section. |
| In Table 2 the definitions of predictive values are too narrow: these terms are used within diagnostic assessments and thus revolve around a test providing a result rather than an individual reporting something. Additionally the definition for AUC does not actually state how AUC is derived, it focuses more on the definition of the ROC (receiver operating characteristic) curve. | Thank you for your detailed review of this table. We aimed to provide an overview of these measures, and given that other studies (cited in the title of the table) provide a much more in-depth consideration and calculation of these methods, we would prefer to keep this table in its current form rather than duplicating. |
| Table 3 – the text indicates that it is about indicators in population-based surveys. This should be reflected in the title of the Table. | Thank you for this suggestion, we opted to revise the text describing this Table. |
| Table 4 provides limited details about the data used and the methods of comparison. For example for Caesarean section the source reference uses both coefficients obtained using linear regression, for multiple studies, and also provides a scatterplot which displays the data. By contrast for RMNCAH is for just one study and each of the four methods of estimation for a number of indicators are reported, which enables simple differences to be derived, though they are not actually derived. | We thank the reviewer for this suggestion. However, we believe that for the purpose of showing a range of indicators and methods of assessing convergent validity of indicators, the table in its current form is sufficient, and avoids too much detail. The intention of this table was not to provide exhaustive details of each individual study. The readers are able to refer to each study mentioned for more information. |
| **Reviewer 4** |  |
| This is a very interesting article; however, there are some aspects that need to be incorporated in some sections in order to improve the manuscript and comprehension, especially regarding methods. | Thank you for your feedback and suggestions, which we have incorporated in the manuscript. |
| Specific Comments:  Abstract: Question posed: the question posed is clear, it should include the purpose of the study.  Introduction: complete and updated, the objectives of the study are clearly stated. | We have added the purpose (objective) of the study in the Abstract. |
| Methods:  Authors mentioned that this paper was developed iteratively, guided by principles for conceptual framework defined by Miles and Huberman (20), they should provide readers a brief description or definition about this framework and how this applies to this paper, not just mention | We thank the reviewer for this suggestion; we have addressed this in the Methods section. |
| They also mention having used a purposive sampling to identify key informants and having finally interviewed 32 respondents from different backgrounds, it is important and interesting having the description of these different backgrounds. | We thank the reviewer for these suggestions. Please note we have published a separate paper with details of the key informant interview findings. We have added information on respondents’ background in brief and provide a reference to the full paper for more detail. |
| Results:  I suggest authors to include a briefly description on finally how many studies were included in this review and a scheme showing the way they were selected and excluded.  Tables and figures: I suggest to include a figure or scheme describing the number of studies included those selected and those excluded, this will help to clarify the reader´s comprehension. | Thank you, we have added this information in the Methods section. We do not present a classic flow chart as we did not attempt a systematic review or systematic scoping review; the selection of studies for Tables 1-4 was purposeful to present a range of studies and examples. |
| In my opinion the heading Key terms in indicator validity line 174 page 6, containing definitions, should be moved to the introduction, or the methods section, this is not part of the results. The same occurs with the text written from line 223-227 in page 8, in my opinion authors discussed their findings, and therefore this should be moved to the discussion section. Some limitations are stated in this section lines 237-242, which in my opinion should be moved to the corresponding section. | Thank you – we moved the overall definition of validity to the Methods section and retained the three specific types of validity found in the field of maternal and newborn health indicators in the Results section.  We opted to retain the limitations of the specific types of validation approaches in the Results section, as these are our findings (from literature and key informant interviews). The overall limitations section of our manuscript is more concerned with how the conceptual framework was developed. |
| Discussion: Complete and comprehensive, see the above comment to be considered with regard the limitations of this study.  Conclusion: adequate. | Thank you. |
| Author’s contribution: Not mentioned | Thank you for noticing this.  The author roles were as follows:  Conceptualization: LB, A-BM, ACM  Methodology: LB, A-BM, KH, LMEV, AM, CH, KS, SA, ACM  Data curation: LB, A-BM, ACM  Formal analysis: LB, A-BM, KH, LMEV, AM, CH, KS, SA, ACM  Visualisation: LB, A-BM, KH, LMEV, AM, CH, KS, SA, ACM  Writing – original draft: LB, A-BM, ACM  Writing – review & editing: LB, A-BM, KH, LMEV, AM, CH, KS, SA, ACM  Funding acquisition: ACM, A-BM |
| Ethics Statements: No ethics approval was required because of the nature of this study. | No ethics approval was required. Key informant interviews were conducted about indicators and not human data or human tissue, we did not require an ethics approval.  The data in the form of interview notes is available open access under the DOI:  <https://doi.org/10.17037/DATA.00001403>  All key informants were asked to review the interview notes and agreed to have their anonymised interview notes included in this open access data file.  A separate paper was published on the key informant interviews, it is available here:  <https://journals.plos.org/plosone/article?id=10.1371%2Fjournal.pone.0224746> |
| Conflicts of interest: "The authors have declared that no competing interests exist." | A competing interests statement is not provided in the Manuscript file, but in the PLOS One submission system. |
| References: This section follows the journal requirements. | Thank you. |
| **Reviewer 5** |  |
| Thank you for the opportunity to review the manuscript. The authors should be congratulated on a well-written and timely paper presenting a range of perspectives on how validity of maternal and newborn indicators is defined and understood by those who develop and use these indicators. The study has important implications for ongoing global efforts to track maternal and newborn health progress. Please see below for specific comments on the manuscript. | Thank you for your thorough review and appreciation of this manuscript. |
| Materials and methods • Line 117: Please reference the key indicator guidance documents used in the review | Thank you, these references have been added. |
| • Line 22: Can the authors please provide further details about the key informants’ backgrounds/expertise on this topic or in MNH? The authors provide some background information in the limitations section of the discussion however it would be useful to have this outlined earlier. Were all key informants’ part of the MoNITOR group? • Was there any pre-testing of the interview guide prior to the 32 key informant interviews? • What was the consent process? • Reference 23: Is this paper accepted for publication or under review? Please update reference. • Line 127: What written materials and relevant publications were received from the respondents after the interview? How were these relevant to the interviews? • Can the authors please clarify the process for the qualitative data analysis? Apologies if I have overlooked this. Was the analysis undertaken by the same person who undertook the interviews? | We thank the reviewer for these suggestions. Please note we have published a separate paper with details of the key informant interview findings. We have added information on the methods of the key informant interviews and provide a reference to the full paper for more detail. |
| Results • Line 223: The authors state that key informants noted that a substantial portion of recent work on assessing criterion validity has focused on indicators of care coverage. It would be useful to know what proportion of the key informants identified this to be the case. | Thank you for this suggestion. However, given the semi-structured interview guide (Supplementary material to the paper referenced here), we did not pose this exact question to all the key informants, therefore only those that found it important/relevant enough to mention spontaneously would have done so. Given the qualitative approach to these interviews, we do not find it appropriate to provide a quantitative summary measure of frequency. We support this statement with our review of the literature and feel this finding, based on these two data sources, is justified. |
| The discussion is well-written, and the conclusion is concise and logical. This is an interesting and useful paper that should be considered for publication following revision of comments. | Thank you. |
| **Reviewer 6** |  |
| In general terms, the present study aims at gathering concepts and themes essential when analysing indicator validity in the context of maternal and newborn health indicators with the goal of reaching high health standards of these two population groups. Personally, I believe the main subject of this original body of work made a substantial contribution in presenting the existing knowledge about indicator validity through a qualitative investigation based on interviews to key informants, literature revision and which had the participation of the MoNITOR group. The results of the investigation are a tool that will allow all stakeholders in maternal and newborn health to reconsider the ongoing work about indicator validity. This work will improve long- term health of pregnant women and their newborns who are a vulnerable group through their life course. | Thank you. |
| Introduction: In this section, reference is made to the background and settings in which this work is developed, objectively contributing to filling the information gap existing when discussing indicator validity in maternal and newborn health. Consequently, the objective of this study was clearly stated in this section. I suggest reviewing the content in parentheses in line sixty-eight. | Thank you, we have removed the parentheses. |
| Materials and methods: This section includes an organized structure in which the study design, sample and type of sample for the selection of key informants are stated. It also explains, in detail, the terms used to conduct the review and it sets the limits of maternal and newborn health indicators. Moreover, it incorporates a definition of concepts. Since this work was developed in four stages, I suggest mentioning the four stages in the first paragraph of the section. | We thank the reviewer for this suggestion, which we address in the first paragraph of the Methods section. |
| I also suggest, moving the interview to key informants to the beginning adding that the interview is part of a qualitative study which main result and methodology were already published. Then, I would list the review and MoNITOR group discussion. I give this advice considering that is the way the rest of the section is organised. The second paragraph begins with the type of sample used to select key informants and, even though it is indicated that the first results of these interviews were previously published in another study, I think it is necessary to add the main details of the methods of such work in this section of this study. | Thank you for this suggestion, we have revised the order of the approaches listed in the Methods section. |
| Ethics Approval: I suggest adding, at the end of Materials and Methods, ethical aspects of the study, such as whether the researchers had informed consent of key informants and why they did not present the protocol before the ethics committee. | No ethics approval was required. Key informant interviews were conducted about indicators and not human data or human tissue, we did not require an ethics approval.  The data in the form of interview notes is available open access under the DOI:  <https://doi.org/10.17037/DATA.00001403>  All key informants were asked to review the interview notes and agreed to have their anonymised interview notes included in this open access data file.  A separate paper was published on the key informant interviews, it is available here:  <https://journals.plos.org/plosone/article?id=10.1371%2Fjournal.pone.0224746> |
| Results: The results are in accordance with the objects of the study, but in the case of the review the results are not complete because they do not inform the number of studies obtained through the research nor the studies selected. I suggest completing with these pieces of information. | We thank the reviewer for this comment, which has been addressed in the Methods section. |
| The first paragraph of this section mentions Table S2 (line 168), but in the annexed material there is only a Table S1. Line 297 mentions S1, I suggest checking if this Table is indeed Table 1. | Thank you for noticing, this was a typo and corrected to “S1 Table”. |
| Discussion: This section discusses the study’s main results. Some aspects are discussed in the section Results, for instance, when limitations of the use of the denominator in indicator validity are mentioned. | Nothing to address. |
| Conclusion: It focusses in the objectives of the study, highlighting the main recommendations of the study. | Thank you. |
| Bibliographic references:  • I suggest revising the references and writing them according to Vancouver style. • I suggest to add the “doi” to all the citations. • Review the reference number 20, seems to be have two references together. • Standardize the name of the journals. Some journal´s names are written completely and others, abbreviated. • Please, be sure you have the publication tittle name of all your citations, not only the link, and the date of access. | Thank you, we have checked the citation style and ensured that it follows the journal guidance.  Reference 20 in the original manuscript has been reviewed and corrected.  When we cite websites, we provide the link and the access date. |
