# Supplementary material for: What is meant by validity in maternal and newborn health measurement? A conceptual framework for understanding indicator validation
Source: PLoS One. 2020 May 29;15(5):e0233969. doi: 10.1371/journal.pone.0233969 (PMC7259779; doi:10.1371/journal.pone.0233969)
Supplement: Attachment — Submitted filename: 20200515_Response to reviewers_R2_FINAL.docx [file pone.0233969.s006.docx]

Dear Dr Sacks	May 15, 2020

Thank you very much for considering our manuscript. Your and the reviewers’ suggestions on how to strengthen it are very appreciated. Below we respond to the feedback point-by-point.

| **Comment** | **Response** |
| --- | --- |
| **Academic editor** |  |
| Can you specify who the key informants were. I know there is reference to another paper will full methods, but it would be good to summarize in a sentence or so here. | Thank you for noting this. We have provided this additional clarification:  “First, AM, A-BM and LB drew up a list of potential key informants through discussion and with input from the MoNITOR co-chairs. The list was further expanded using snowball methods to encompass qualitative and quantitative measurement experts on the various types of maternal and newborn indicators (health system and input, care access and availability, quality of care and safety, coverage and outcomes, and health impact).  The final sample of 32 key informants interviewed included 22 measurement experts based in academic institutions, four from funders operating in the space of maternal and newborn health, two from United Nations agencies, two from implementing agencies, and two from data collection organisations.” |
| Can you clarify if no ethical approval was sought, or if it was exempted because considered to be non human subjects research? The paper includes a quote, so even though it's not sensitive personal information, many ethical review boards would want to review a protocol that includes interviews. | Thank you for the question. We did not seek ethical approval, and we clarified this in the manuscript. We sought permission from all respondents before including their responses in the data which were made available through an open-access data repository. The data archiving was reviewed and approved by the LSHTM Ethics committee. |
| Can you address in the discussion the additional challenge with measuring newborn quality of care because of who is reporting (if the baby is taken out of the room, the mother can't report and the health worker may not record etc etc.) since newborns have limited verbal communication as opposed to measures for women who can self-report. | Thank you for this suggestion. We have added this important point in the last paragraph of the Results section, where it fits with other considerations of measurement of quality of care. |
| **Reviewer 1** |  |
| I reviewed comments I made on the earlier draft of this paper. Compared to that early draft, this paper is much, much better. I appreciate the authors taking into consideration the advise shared and incorporating. | We thank the reviewer for their thorough and constructive feedback. |
| The paper is a much better ps thaper, and I don't have any additional comments. I like their use of examples of indicators within each category of validity. It would have been useful to then show a table with some indicators that meet all validity criteria, as this would help the reader think about how to evaluate an indicator they choose to use with the aim of getting the most valid indicators possible (gold standard). It would be a useful contribution if the authors conclude with an example of a few indicators that are like 'gold standard' MCH indicators because they meet various validity criteria and all researchers are encouraged to include these in their studies. This would be similar to how demographics indicators always include age and gender. | Thank you for this suggestion. We discussed this during the initial stages of this research, but found that we cannot do justice to such a “table” or overview, either in this manuscript or within the limits of this research study. There are two main reasons:   1. A thorough appreciation of the literature describing the validity performance of the dozens of maternal and newborn indicators requires data collection through systematic reviews. We tried this for the indicator of “skilled birth attendant” in a separate [manuscript](https://www.ncbi.nlm.nih.gov/pubmed/31139455); the type of effort required to compile this type of evidence for any number of indicators was not possible within this current research project. 2. Published studies provide an appreciation of the performance of indicators for criterion and convergent validity. However, as we attempted to highlight in this manuscript, when selecting indicators to use, consideration also needs to be given to construct validity. What is the researcher or programme aiming to evaluate/capture (phenomenon/construct)? In which setting? What alternative data are available? None of these questions can be satisfactorily addressed in a simple table. Such work is currently being conducted by the WHO through implementation guidance and identification of gaps in indicator validation (<https://www.who.int/data/maternal-newborn-child-adolescent/monitor>). |
| **Reviewer 2** |  |
| Overall, the authors appear to have satisfactorily addressed the comments and the manuscript is clearer and much improved. No further revisions are suggested at this time. | We thank the reviewer for their comments during this peer-review process. |
| **Reviewer 3** |  |
| The relationship between validity and reliability raised by reviewer 1 is not adequately addressed. The authors assert that ‘theoretically an indicator can be valid but unreliable despite this being counterintuitive and uncommon’. No basis for this assertion is given. Streiner et al (2015) in their monograph “Health measurement scales: a practical guide to their development and use” (5th edition) explain that reliability places an upper limit on validity. A previous article by Streiner and Norman is cited (39) in support of Figure 2. I do not have access to this article. However Streiner et al (p164) say “We have argued (Streiner and Norman, 2006) that the other terms ignore the equally important part of the denominator, “Subject variability”. . . .if two raters place all of their students in the ‘above average’ category, and do so again when asked to re-evaluate the students one week later, their repeatability, reproducibility, consistency and agreement will all be perfect, but the reliability will be zero, because there is no true difference among those being rated.” This example illustrates why the assertion is not well-founded. | Thank you for your comment. We would respectfully like to note that Reviewer 1 was satisfied with our response to this suggestion.  We have added additional justification and citations to support this statement, with specific references to population health indicators, rather than other disciplines. |
| Thank you for moving the definition of validation. However it is at the end of the materials and methods section, after numerous uses of the term. An earlier indication of the definition would be preferable. | Thank you for this suggestion. We moved the definition of validity into the last section of the Introduction, immediately following the first use of the term in the manuscript. |
| In response to my comment on Table 3 it was indicated that “we opted to revise the text describing this Table”. However the only revision I see is deletion of reference to population-based surveys! | Thank you for noting this, that is correct. Your original comment stated that “Table 3 – the text indicates that it is about indicators in population-based surveys. This should be reflected in the title of the Table.” We corrected the following text in the manuscript to say “We present examples of pre-specified cut-offs provided by studies assessing validity of indicators based on women’s recall (Table 3).”  We believe we addressed the discrepancy between the title of Table 3 and the text describing Table 3. We did not change the title of Table 3, rather we amended the text which describes Table 3, to be “based on women’s recall” which is more accurate than “based on population-based surveys”. |
